# Detection and diagnosis of periodontal conditions amenable to prevention

**DOI:** 10.1186/1472-6831-15-S1-S5

**Published:** 2015-09-15

**Authors:** Philip M Preshaw

**Affiliations:** 1 School of Dental Sciences and Institute of Cellular Medicine, Newcastle University, Newcastle upon Tyne, NE2, 4BW, UK

## Abstract

Gingivitis and chronic periodontitis are highly prevalent chronic inflammatory diseases. Gingivitis affects the majority of people, and advanced periodontitis is estimated to affect 5-15% of adults. The detection and diagnosis of these common diseases is a fundamentally important component of oral health care. All patients should undergo periodontal assessment as part of routine oral examination. Periodontal screening using methods such as the Basic Periodontal Examination/Community Periodontal Index or Periodontal Screening Record should be performed for all new patients, and also on a regular basis as part of ongoing oral health care. If periodontitis is identified, full periodontal assessment is required, involving recording of full mouth probing and bleeding data, together with assessment of other relevant parameters such as plaque levels, furcation involvement, recession and tooth mobility. Radiographic assessment of alveolar bone levels is driven by the clinical situation, and is required to assess bone destruction in patients with periodontitis. Risk assessment (such as assessing diabetes status and smoking) and risk management (such as promoting smoking cessation) should form a central component of periodontal therapy. This article provides guidance to the oral health care team regarding methods and frequencies of appropriate clinical and radiographic examinations to assess periodontal status, to enable appropriate detection and diagnosis of periodontal conditions.

## Introduction

Periodontal diseases are highly prevalent chronic inflammatory conditions that affect the supporting tissues of the teeth. In broad terms, and of most relevance to the global community, these include gingivitis (i.e. plaque-induced gingivitis) and chronic periodontitis. This paper will review the methods for detection and diagnosis of gingivitis and chronic periodontitis, these being periodontal lesions that are amenable to prevention, and will take the form of a narrative review.

## Pathogenesis of periodontal conditions

Gingivitis and chronic periodontitis are highly prevalent, chronic inflammatory conditions. The last 40-50 years have witnessed a transformation in our understanding of the pathogenesis of these common conditions. The role of bacterial plaque in initiating gingival inflammation is unquestioned, and was first demonstrated in experimental gingivitis studies in the 1960s [1]. Much of the 1960s and 1970s were dominated by treatment concepts that focussed exclusively on removal of calculus and “necrotic” root cementum that was believed to be infected by bacterial toxins such as lipopolysaccharide (LPS). However, ongoing research in the 1980s and 1990s resulted in increasing awareness of the importance of the inflammatory host response as an important determinant of risk for disease [2,3]. As technological advances have been made in the fields of microbiology, immunology and inflammation, we now recognise that inflammation is at the heart of the destructive responses that lead to the tissue breakdown that we recognise clinically as gingivitis and periodontitis. Accumulation of plaque bacteria in the subgingival environment results in diffusion of bacterial products and toxins across the junctional epithelium into the host tissues. As a result, the host mounts an immune-inflammatory response that is characterised by a complex network of cellular and molecular interactions in the host tissues. The complexities of these interactions have been described in detail [4,5] and our understanding of these mechanisms is likely to change and evolve with further research and technological innovations. The underlying principle is that the immune/inflammatory response to the subgingival biofilm varies greatly between individuals, and is controlled at a number of regulatory (e.g. pro- and anti-inflammatory cytokines, feedback loops), genetic, and epigenetic levels.

Inflammation is intended to defend the host against the bacterial challenge, but prolonged and/or excessive inflammation results in tissue damage. Periodontal disease is now regarded as a non-resolving chronic inflammation that is initiated and perpetuated by the subgingival bacteria, but which is ineffective in removing the bacteria, and which, over time, leads to the tissue damage that we recognise as periodontitis [6,7]. It is important to note that gingivitis is a reversible condition, if the inflammation can be controlled. This normally is achieved by improving oral hygiene and reducing the bacterial biofilm [8,9]. If the biofilm is not controlled, gingivitis will persist, and in some patients, may progress to periodontitis [10]. Periodontitis is differentiated from gingivitis by the progressive breakdown of periodontal ligament fibres ("loss of attachment") resulting in increased probing depths, and resorption of alveolar bone, and the tissue damage that occurs is largely irreversible.

Our current understanding of periodontitis pathogenesis is that susceptibility to disease ("disease” being the clinical manifestations that result from the persisting inflammation and tissue breakdown) appears to be largely determined by the nature of the inflammatory host response. In the classic experimental gingivitis studies of the 1960s, it was noted that inflammation developed more rapidly in some individuals as compared to others, even though plaque accumulation was similar [1]. More recently, carefully controlled experimental gingivitis studies have revealed the same finding, namely that the intensity of gingival inflammation varies widely between individuals following plaque accumulation, suggesting that susceptibility to disease varies between individuals due to differences in the inflammatory host response [11], rather than being entirely due to differences in the amount and/or composition of the bacterial plaque.

The importance of the host response in determining susceptibility to chronic periodontitis was clearly documented in carefully conducted longitudinal observational studies of tea plantation workers in Sri Lanka. These individuals had no access to dental care, did not routinely use conventional oral hygiene products, and presented with generalised plaque and calculus deposits. Yet, within this population, around 11% were considered to be stable, with no evidence of progression of periodontitis, another group (81%) demonstrated moderate progression of periodontitis, and 8% showed rapid disease progression [12]. Longitudinal studies of patients on long term periodontal maintenance programs have also reported that a small subgroup of patients appear to be particularly susceptible to disease, with periodontitis progression occurring despite ongoing maintenance care [13,14].

## Prevalence of periodontal conditions

Plaque-induced inflammatory periodontal conditions are highly prevalent. However, prevalence estimates for periodontitis have changed greatly over the years, as a result of changes in the methods used to detect the presence of disease in epidemiological studies [15]. Thus, in the 1950s and 1960s, the use of periodontal indices such as the Russell Index (which assumed continuity between gingival and periodontal inflammation) resulted in the presumption that periodontal diseases were virtually ubiquitous, with a sense of inevitability that all adults would develop periodontitis [16]. However, more recent research has suggested that, while gingivitis and mild periodontitis are highly prevalent, advanced periodontitis is not quite as prevalent as previously perceived [16].

Many epidemiological studies have used the CPITN (Community Periodontal Index of Treatment Need) [17] to identify periodontitis. This method has the advantages of being quick and easy to perform, as well as being understood and used throughout the world. On the other hand, limitations include that it assesses probing depth only, and does not provide any information on loss of attachment. Furthermore, it only records the most severe score in each sextant (and therefore does not provide full information about disease extent and severity in advanced cases) [18]. Inherent to the originally described CPITN scoring system was a link between CPITN score and treatment requirement; however, this link is somewhat questionable, and more recent iterations of the scoring system have renamed it as the CPI (Community Periodontal Index), with removal of the “treatment needs” component [15]. Adaptations of the CPI (or CPITN) have also been provided by the American Dental Association and American Academy of Periodontology (the “Periodontal Screening Record”, PSR), and the British Society of Periodontology (the “Basic Periodontal Examination”, BPE) [19-21] (Table 1).

**Table 1:** Basic Periodontal Examination (BPE) scoring codes [19]

Code	Descriptor
0	No pockets >3.5 mm, no calculus/overhangs, no bleeding after probing (*black band completely visible*)
1	No pockets >3.5 mm, no calculus/overhangs, but bleeding after probing (*black band completely visible*)
2	No pockets >3.5 mm, but supra- or subgingival calculus/overhangs (*black band completely visible*)
3	Probing depth 3.5-5.5 mm (*black band partially visible, indicating pocket of 4-5 mm*)
4	Probing depth >5.5 mm (*black band entirely within the pocket, indicating pocket of 6 mm or more*)
*	Furcation involvement

Note: both the number and the * should be recorded if a furcation is detected - e.g. the score for a sextant could be 3* (e.g. indicating probing depth 3.5-5.5 mm plus furcation involvement in the sextant). The highest score is recorded for each sextant.

A methodological concern in periodontal epidemiology is whether to use partial mouth or full mouth recordings of periodontal status. Clearly, partial mouth recordings are quicker to undertake than full mouth recordings, and this may be important when screening a large number of individuals. However, it is well recognised that partial mouth recordings result in underestimation of the prevalence of disease [22-25]. Accordingly, it has been noted that the CPI results in an underestimation of periodontal disease prevalence, though, on the other hand, it has been recognised as being well suited for identifying individuals who are (and who continue to be) periodontally healthy [22].

A major consideration is the case definitions that are used to denote a patient as a periodontitis case. Clearly, the calculated prevalence of periodontitis is fundamentally dependent on the case definition used to assign the diagnosis of periodontitis [26]. Case definitions for periodontitis to be used in epidemiological studies have been proposed (Table 2) [27-29]. Clearly, while it is important to define the criteria for assigning a case of periodontitis in epidemiological studies, more comprehensive information is likely to be required by clinicians when assessing the presence, extent and severity of periodontitis in their individual patients.

**Table 2:** Case definitions for denoting periodontitis in epidemiological studies

Authors/context	Case severity	Case definition
Tonetti & Claffey, 2005. Consensus report of the 5^th^ European Workshop on Periodontology [28]	Mild/incipient cases	Presence of proximal attachment loss of ≥ 3 mm in ≥ 2 non-adjacent teeth
	Severe cases	Presence of proximal attachment loss of ≥ 5 mm in ≥ 30% of teeth present

Page & Eke, 2007. US Centre for Diseases Control and Prevention (CDC) and American Academy of Periodontology (AAP) [27,29]	Mild periodontitis	Two or more interproximal sites with attachment loss ≥ 3 mm and two or more interproximal sites with probing depths ≥ 4 mm, not on the same tooth, or one site with probing depth ≥ 5 mm
	Moderate periodontitis	Two or more interproximal sites with attachment loss ≥ 4 mm, not on the same tooth, or two or more interproximal sites with probing depths ≥ 5 mm, not on the same tooth
	Severe periodontitis	Two or more interproximal sites with attachment loss ≥ 6 mm, not on the same tooth, and one or more interproximal sites with probing depth ≥ 5 mm

When taking into consideration the large number of national studies of periodontal epidemiology that have been conducted [16], and bearing in mind the different methodological techniques and case definitions used to define a case of periodontitis, it is generally estimated that 5-15% of adults in populations that have been studied have severe chronic periodontitis (as evidenced by having, for example, at least one periodontal pocket of ≥ 6 mm) [16]. Prevalence estimates for moderate periodontitis (e.g. maximum probing depths of 4-6 mm) are less precise, but are probably within the range of 30-50% of adults [16]. There is a shortage of precise data regarding the prevalence of gingivitis, which is generally considered to be very high, probably affecting the great majority (e.g. >75%) of people.

A major limitation in our current understanding is that we do not have the ability to be able to recognise which sites with gingivitis will progress to periodontitis, or, which sites with periodontitis will progress further. Whereas it may be feasible to conduct studies in experimental animals to study the transition from gingivitis to periodontitis, ethical concerns preclude the same sorts of experiments from being performed in humans. Furthermore, it has also been demonstrated (in experimental animals) that even longstanding gingivitis does not necessarily progress to periodontitis [30]. It is also not possible to determine, with accuracy, which sites are undergoing progressive tissue breakdown. Instead, we rely on detecting the signs of previously occurring tissue damage, through the use of periodontal probes (to detect loss of attachment and increased probing depths) and radiographs (to detect the historical occurrence of alveolar bone destruction).

## Risk assessment

It is well recognised that a number of environmental exposures significantly increase the risk for periodontitis. Preeminent among these are smoking and diabetes. Smoking has long been recognised as a risk factor for periodontitis, with a 1.4 to five-fold increased relative risk for periodontitis among smokers compared to non-smokers [31]. It has also been reported, based on data from the National Health and Nutrition Examination Survey III (NHANES III) conducted between 1988 and 1994, that smoking (current smoking or former smoking) may be responsible for approximately half of periodontitis cases among adults in the United States, with the implication being that a large proportion of chronic periodontitis cases may be preventable through prevention and cessation of smoking [32]. In support of this, smoking cessation has been associated with improved outcomes of periodontal therapy [33], and should form a central component of the periodontal management of all smoking patients, including patients with and without periodontitis.

Diabetes is also recognised as a major risk factor for periodontitis, with poorly controlled diabetes increasing the risk for periodontitis approximately 3-fold [34]. The precise mechanisms by which diabetes increases the risk for periodontitis are not yet fully characterised, but almost certainly relate to modified inflammatory and immune mechanisms which increase the susceptibility to the condition [35]. The level of glycaemic control is important in determining risk; thus, people with well controlled diabetes are at minimal/no increased risk for periodontitis compared to those who do not have diabetes, whereas people with poorly controlled diabetes are at much greater risk [34].

Given the importance of factors such as smoking and diabetes in risk for periodontitis, assessing risk should form a standard component of periodontal assessment. This is undertaken as part of the history and examination, and every effort should be made to reduce or eliminate risk factors as part of periodontal therapy. A systematic approach should be employed when obtaining the history from the patient. With regards to the main systemic risk factors of smoking and diabetes, some key questions to be asked include:

• do you smoke, if so, for how many years, and how many cigarettes per day?

• if you previously smoked, when did you quit? And, before then, for how many years did you smoke, and approximately how many cigarettes per day?

• with regards to your diabetes, how would you rate your level of diabetes control (e.g. good/poor)? Do you know your most recent HbA1c (glycated haemoglobin) measurements?

In the case of a patient with poorly controlled diabetes and advanced periodontitis, it may also be useful to liaise with the patient's medical clinician so that they can also emphasise the importance of improving periodontal health (and also of maximising glycaemic control) as part of overall management.

To summarise current knowledge, it is known that the accumulation of the subgingival biofilm results in a cascade of immune and inflammatory responses which leads to development of gingivitis, and in some cases, periodontitis. The onset and rate of progression of periodontitis vary greatly from person to person, and multiple factors (microbiological, environmental, immune and inflammatory) interact to determine individual susceptibility to disease. Examination of a patient with periodontitis must, therefore, not only focus on detailed assessment of the clinical condition, but must also include assessment of risk for disease.

## Detection of periodontal conditions

Detection of periodontal conditions is complex and requires a high degree of skill, both as a communicator to understand the patient's problems, and as a clinical operator to detect disease. Key factors of the clinical examination will now be described.

### 1. Clinical examination and periodontal probing

The examination of the gingival and periodontal tissues should occur in a logical sequence. Most operators begin with a visual inspection of the gingival tissues to assess (somewhat subjectively) the presence or absence of gingival inflammation (by assessing the colour and degree of swelling of the tissues) as well as an initial assessment of the level of oral hygiene (assessing plaque and calculus levels). Following this, assessment of probing depths occurs. The first decision to make is the choice of periodontal probe. For epidemiological studies, the World Health Organisation (WHO) Community Periodontal Index (CPI) probe may be used, to assign a score to each sextant, dependant on the most affected site (as shown in Table 1). The WHO CPI probe is specifically designed for this purpose, with a 0.5 mm ball tip (to minimise penetration of the probe into the soft tissues and also to help in the detection of calculus), a black band between 3.5 and 5.5 mm, and rings at 8.5 and 11.5 mm. However, for the individual patient in clinical practice, more detailed information may be required, particularly for patients with periodontitis, so that the precise probing depths throughout the dentition are recorded. A variety of periodontal probes are available for this purpose, including manual probes (e.g. Williams, UNC PCP-15) or computerized periodontal probes (e.g. Florida probe). On the other hand, recording a full periodontal charting for periodontally healthy patients at every visit would be excessively time consuming and laborious, and may even deter patients from attending the dentist. Recommendations regarding periodontal probing are given in Table 3, according to (in broad terms) the type of patient that is being assessed.

**Table 3:** Recommendations for assessment of periodontal status by means of periodontal probing

Type of patient	Type of probe	When to use	Rationale
Patients who do not have periodontitis	WHO CPI	At every check-up visit (at least annually)	The CPI/BPE/PSR is known to result in underestimation of periodontal disease severity in patients with periodontitis. However, it is well suited for identifying individuals who do not have periodontitis. Therefore, on the basis that it is relatively quick and easy to perform, it should be used to screen patients for the absence of periodontitis on a regular basis as part of their routine “check-up” visits.
Patients with periodontitis (newly diagnosed)	UNC PCP-15	Pre-treatment to record baseline periodontal status. Post-treatment (approximately 3 months) to assess response to initial therapy and determine future treatment need	For patients with periodontitis (indicated by code 3 or code 4 of CPI/BPE/PSR), then more detailed periodontal charting is recommended. For a patient with any code 4 score, then full periodontal charting should be performed to obtain a pre-treatment record (6 sites per tooth). A post-treatment charting should be performed after the initial (non-surgical) therapy, typically at 3 months post-initial treatment, to assess the response and determine next steps (e.g. more non-surgical therapy, surgical intervention).
Patients with treated periodontitis, who are now in the maintenance phase of treatment (supportive periodontal care)	UNC PCP-15	Annually (although more frequent probing may be required if concerned about specific sites or teeth, or if there is evidence of ongoing progression)	For patients undergoing periodontal maintenance care, full periodontal charting should be performed (6 sites per tooth) at least annually to assess for evidence of disease progression.

WHO CPI: World Health Organisation Community Periodontal Index probe

UNC PCP-15: University of North Caroline PCP-15 periodontal probe (an example of a manual periodontal probe, other probes may also be used)

The probing force that is used during the clinical examination clearly has the potential to influence the recorded measurements, as does the degree of inflammation in the gingival and periodontal tissues. In general terms, in the presence of inflammation, the probe tip penetrates the base of the junctional epithelium, leading to an overestimation of pocket depth, whereas in the absence of inflammation, the probe tip does not reach the base of the junctional epithelium [36]. For this reason, it is important to note that the measured probing depth does not equate exactly to the true pocket depth, and for this reason, the term “probing depth” (or “probing pocket depth") should be used (as opposed to “pocket depth"). With regards to the optimal probing force, this should be selected to achieve a measurement of probing depth that is as accurate as possible (i.e. not significantly over- or under-estimating pocket depth) while also being as comfortable as possible for the patient (recognising that inflamed tissues in which the probe penetrates the junctional epithelium are more likely to be painful on probing compared with non-inflamed tissues). It is generally recognised that around 0.20-0.25 N is the optimal probing force (equivalent to approximately 20-25 g) [36]. However, for the clinician, it is difficult to assess this amount of force, which has been described alternatively as the pressure required to blanch the tissues when the probe point is placed under the thumbnail, or, as the pressure required to depress the skin on the pad of the thumb by about 1 mm.

When undertaking a screening evaluation using CPI/BPE/PSR, a systematic approach should be adopted, and each sextant should be fully assessed before moving on to the next sextant. There is no right or wrong sequence of probing; the main issue is to be systematic so that no areas are missed. Some practitioners follow a sequence as follows: upper right, upper anterior, upper left, lower left, lower anterior, lower right. Others may prefer to go from right to left on all passes.

When recording full mouth periodontal probing depths (in the case of a patient with periodontitis), a systematic approach is again used. A common approach is as follows:

• buccal surfaces of upper arch (from right to left)

• palatal surfaces of upper arch (from left to right)

• buccal surfaces of lower arch (from right to left)

• lingual surfaces of lower arch (from left to right)

Probing depth measurements are recorded at 6 sites per tooth (mesio-buccal, mid-buccal, disto-buccal, mesio-palatal, mid-palatal, disto-palatal). Bleeding on probing (BOP) should also be recorded as present or absent at each site following probing, and provides (somewhat limited) information about the level of inflammation in the periodontal tissues. Whereas presence of BOP at isolated sites is not a particularly good indicator of “active” inflammation or risk of disease progression [37], absence of BOP is a reasonably good indicator of periodontal health and tissue stability [38,39]. On the other hand, persistent BOP at sites that also demonstrate increasing probing depths is a strong indicator of risk for future progression of disease [40]. Furthermore, in patients undergoing periodontal maintenance care, persistent bleeding on probing at successive maintenance visits is a strong indicator of risk for ongoing disease progression [41].

### 2. Radiographic assessment

For patients with evidence of periodontitis, radiographic assessment is essential to provide information regarding the pattern and extent of alveolar bone loss. Guidance is provided by relevant authorities in different countries around the world, and for the purpose of this paper, the guidance issued by the Faculty of General Dental Practice (UK) will be described [42]. In broad terms, the use of radiography is driven by, and is secondary to, the results of the clinical examination. Recommendations adapted from those provided by the FGDP are presented in Table 4.

**Table 4:** Recommendations for radiographic assessment of periodontal status*

Scenario	Recommendation
Patient in whom clinical examination indicates that it would be useful to assess all their teeth and their periodontal support	Full assessment of all teeth and alveolar bone status can be achieved by: - an optimal quality panoramic radiograph alone - an optimal quality panoramic radiograph with supplementary periapical radiographs depending on the clinical situation - a complete series of periapical radiographs When determining which technique to use, consider the clinical situation, the required image quality, and the relative dose-benefit based on the radiographic equipment available.
Suspected periodontal/endodontic lesion	A periapical radiograph is indicated.
Specific periodontal scenario:patient with generalised probing depths of ≤ 3-4 mm	This level of probing depth is generally indicative of periodontal health. Radiographs are usually not indicated to routinely assess alveolar bone status in this situation.
Specific periodontal scenario: patient with generalised probing depths of ≈ 4-5 mm (e.g. CPI/BPE/PSR scores of code 3)	This level of probing depth is generally indicative of mild/moderate periodontitis. Alveolar bone levels may be adequately assessed by horizontal bitewings taken for routine caries assessment, supplemented by intraoral periapicals for selected teeth depending on the clinical situation. Alternatively, full assessment of all teeth and alveolar bone status may be undertaken as described above, if clinically indicated.
Specific periodontal scenario:patient with generalised probing depths of ≈ 6 mm or more (e.g. CPI/BPE/PSR scores of code 4)	This level of probing depth is generally indicative of advanced periodontitis. Full assessment of all teeth and alveolar bone status is indicated as described above. As an alternative, some authors advocate the use of vertical bitewing radiographs, supplemented by periapical views, e.g. for selected anterior teeth.
Cone beam computed tomography (CBCT)	Not indicated as a routine method for imaging alveolar bone levels as part of periodontal assessment. If CBCT images are obtained for other purposes, however, and they include the teeth, it is important that assessment of alveolar bone support is included in the radiographic report.

* Adapted from the 2013 UK Faculty of General Dental Practice guidelines “Selection Criteria for Dental Radiography” [42]. Note: whenever periapical radiographs are obtained, a paralleling technique should be used.

Every effort should be made to minimise radiation dose. Therefore, available radiographs that have been taken for other purposes (e.g. caries diagnosis) should be utilised, if possible, to aid in the assessment of alveolar bone levels. Paralleling techniques should be used for intraoral periapicals, and attempts made to position sequential radiographs reproducibly over time to allow for better detection of changes in alveolar bone levels that may occur. There is no clear evidence to support any recommendations regarding the frequency of radiographs taken for periodontal assessment, other than to say that decisions regarding radiographs should be driven by the clinical findings. Thus, in a patient with a past history of periodontitis that has been treated and stabilised, and who is now in the maintenance phase of periodontal care, if there is no evidence of disease progression (e.g. as evidenced by increasing probing depths), then there is no indication to take further radiographs for periodontal assessment.

Regarding radiation dose, this appears to be less (when using modern panoramic machines) with a panoramic radiograph plus a small number of supplementary periapical radiographs (taken according to the clinical situation), compared to a full-mouth series of periapical radiographs [42]. Furthermore, with modern panoramic machines, image quality is such that no additional periapical radiographs may be required. For these reasons, there is a trend to move away from exposing full mouth series of periapical images. Therefore, when using modern panoramic machines, it is recommended that a panoramic radiograph is sufficient to assess alveolar bone status, but this may be supplemented by selected periapical radiographs according to the specific clinical situation.

Whenever radiographs are obtained, a written report should be entered into the clinical notes. This should typically include factors such as:

• teeth present (including unerupted teeth)/teeth missing

• bone loss, including pattern (e.g. horizontal, regular, irregular) as well as extent (usually expressed as a proportion or percentage of the root length)

• presence of any specific vertical bone defects

• presence of calculus (supra- and subgingival)

• apical pathology

• caries and enamel lucencies

• ledges/overhangs of restorations

• any other findings or pathology

### 3. Other investigations that form part of periodontal assessment

Periodontal probing to assess probing depths and bleeding on probing, together with radiographic assessment, remain the cornerstone of periodontal assessment. Additional measures that may be recorded, depending on the clinical situation are summarised below.

#### Recession and loss of attachment

Probing depths alone can sometimes be misleading in terms of assessing the cumulative effects of periodontal tissue breakdown. For example, a patient with a history of periodontitis who has been successfully treated may present with shallow probing depths yet with generalised gingival recession (indicating widespread loss of periodontal tissue support that may not be suggested by inspection of the probing depth data alone). It may also be important to measure recession in cases of localised gingival recession. Therefore, while not essential for all patients, the measurement of recession adds to the clinical information that is obtained, and may influence treatment decisions. Probing depth measurements can be summed with recession measurements to obtain loss of attachment:

• Probing depth (*x* mm) + recession (*y* mm) = loss of attachment (*x* + *y* mm)

#### Tooth mobility

Loss of attachment and alveolar bone loss can result in increased tooth mobility. This should be assessed using rigid instruments (e.g. the ends of dental mirror handles) and a score allocated to affected teeth. Several scoring systems for tooth mobility have been proposed, but one that is in common use is shown below [43]:

• Grade I: mobility in excess of physiological mobility ("physiological mobility” is usually considered to be < 0.2 mm in a horizontal direction), but less than 1 mm in a horizontal direction

• Grade II: horizontal mobility > 1 mm

• Grade III: mobility of the crown in a vertical direction

#### Furcation involvement

Progression of periodontitis around multi-rooted teeth may result in horizontal loss of attachment into the furcation area. This should be assessed as part of routine periodontal assessment, bearing in mind the anatomy of multi-rooted teeth. Ideally, a curved furcation probe (e.g. the Nabers probe) should be used for this purpose. In maxillary molars, there are usually 3 roots, and therefore 3 furcations to assess (buccal, mesio-palatal, disto-palatal). In mandibular molars, there are usually 2 roots, and therefore 2 furcations to assess (buccal and lingual). The two main classification systems for assessment of furcations are those proposed by Glickman in 1953 [44] and Hamp in 1975 [45], as shown in Table 5.

**Table 5:** Furcation classification scoring systems

**4-point furcation scoring system proposed by Glickman, 1953**[44]	
Grade 1 furcation	Incipient furcation involvement in which there is pocket formation into the “flute” of the furcation, but no horizontal loss of attachment into the furcation itself
Grade 2 furcation	Loss of attachment into the furcation, but not completely through to the opposite side of the tooth, i.e. is a cul-de-sac furcation involvement
Grade 3 furcation	Horizontal “through-and-through” involvement in which the lesion extends across the entire width of the furcation
Grade 4 furcation	Same as a Grade 3 furcation, but with gingival recession that has rendered the furcation region clearly visible on clinical examination

**3-point furcation scoring system proposed by Hamp et al, 1975**[45]	

Grade 1 furcation	Horizontal loss of attachment into the furcation of < 3 mm (approximately 1/3 the tooth width)
Grade 2 furcation	Horizontal loss of attachment into the furcation of > 3 mm (or approximately 1/3 the tooth width), but does not pass completely through the furcation, i.e. is a cul-de-sac furcation involvement
Grade 3 furcation	Horizontal “through-and-through” involvement in which the lesion extends across the entire width of the furcation

#### Plaque levels/oral hygiene

Given that the subgingival biofilm plays a fundamental role in initiating and perpetuating the inflammation that leads to the clinical signs that we recognise as gingivitis and periodontitis, and also that plaque control is the vehicle by which we aim to control inflammation, assessment of plaque and oral hygiene should form a standard component of periodontal assessment. It is also very important for patients to understand where plaque is accumulating so that they may direct oral hygiene efforts particularly towards those areas of concern. While a large number of plaque index scoring systems have been proposed for research purposes, they are generally not particularly useful for routine clinical practice. Instead, similar to BOP, a dichotomous “present"/"absent” approach can be taken when recording plaque at specific periodontal sites, with the possibility to then calculate a percentage of sites that are covered with plaque. This can be useful for helping to motivate patients towards improving their plaque control. Visualisation of plaque can be further enhanced, as necessary, by using plaque disclosing agents, which may be especially useful for educating children about the importance of improving oral hygiene.

#### Sensibility testing

In some cases, it is useful to perform sensibility testing as part of the periodontal assessment, for example, in cases of suspected periodontal/endodontic lesions. Sensibility should be assessed by a minimum of two independent methods, e.g. cold test (for example ethyl chloride) and electric pulp testing. Results should be recorded in the patient notes.

#### Occlusion

It may be necessary to assess for any evidence of fremitus, occlusal trauma or occlusal interferences. Occlusal trauma can be classified as primary occlusal trauma and secondary occlusal trauma. The reasons for occlusal interferences are diverse, and can include tooth/arch relationships, developmental aspects, or iatrogenic factors. Primary occlusal trauma is said to occur in cases that are periodontally healthy, and may result in increased tooth mobility, widening of the periodontal membrane space, and tenderness, but which does not lead to periodontal tissue breakdown. Secondary occlusal trauma occurs in teeth with pre-existing periodontitis, and may exacerbate periodontal tissue breakdown.

## Assessment of periodontal status in children

Gingivitis is highly prevalent in children, and periodontitis may also be evident (including both chronic periodontitis and aggressive periodontitis) [46]. A joint working group involving the British Society of Periodontology and the British Society of Paediatric Dentistry developed “Guidelines for Periodontal Screening and Management of Children and Adolescents Under 18 Years of Age” [47]. Early detection of periodontal diseases in children and adults is fundamentally important to enable accurate diagnosis, and implementation of correct preventive and treatment approaches. At the same time, there are some challenges associated with periodontal screening of children, such as cooperation (in the case of very young children) and also increased probing depths (false pockets) associated with the mixed dentition stage and partly erupted teeth. It is recommended that assessment of periodontal status should be started at 7 years of age [47], as periodontal problems below this age are very rare, and index teeth are often still unerupted. From age 7 onwards, a simplified Basic Periodontal Examination (BPE) should be performed at 6 index teeth:

FDI 16 11 26

FDI 46 31 36

The scoring system for the BPE in children and adolescents is the same as that used in adults (Table 1), except that in children of 7-11 years old, only the BPE codes of 0, 1 and 2 should be used. For children and adolescents in the age range 12-17 years old, the full range of BPE codes should be used.

## Diagnosis of periodontal conditions

The periodontal diagnosis is a summation of the information from the medical and dental histories, combined with the findings of the clinical and radiographic examination. By its very nature, the diagnosis can be regarded as the clinician's “best guess” as to what condition or disease the patient has [48]. In broad terms, and with regards to plaque-induced periodontal conditions, the diagnosis is typically health, gingivitis, or chronic periodontitis. The most recent internationally accepted classification of periodontal conditions was published in 1999 [49], and is summarised in Table 6.

**Table 6:** Current classification of periodontal conditions*

Gingival diseases and conditions (including plaque-induced gingivitis)
Chronic periodontitis
Localised aggressive periodontitis
Generalised aggressive periodontitis
Periodontitis as a manifestation of systemic diseases
Necrotising ulcerative gingivitis and necrotising ulcerative periodontitis
Abscesses of the periodontium (including gingival and periodontal abscesses)
Combined periodontal/endodontic lesions
Developmental/acquired conditions

*Based on Armitage 1999 and 2004 [48,49]

Whereas this classification system is now in widespread use (and therefore described in this paper), it is important to note that problems and difficulties in its implementation have been identified [50,51]. These primarily result from the fact that periodontitis is a complex disease that has a multi-factorial aetiology but which has a common end-point (loss of attachment and alveolar bone loss). It is beyond the scope of this article to consider these issues in more detail, however.

Assigning a diagnosis is frequently very challenging, and requires an assimilation of all the available evidence and findings. Even experienced clinicians often struggle to assign a diagnosis to a particular case, and frequently will also consider multiple differential diagnoses (i.e. the possible diagnoses, usually listed in decreasing order of likelihood). It is also helpful to include assessments of the extent and severity of disease in the diagnosis. This can make the diagnosis somewhat wordy, but this is certainly acceptable, and is also very useful when describing the condition (much more so than simply referring to the “name” of the condition, with no other detail being provided).

As regards the extent of disease, there are no clear “rules” on what constitutes a localised as opposed to a generalised case in the context of gingivitis or chronic periodontitis. It has been suggested that if > 30% of the teeth are affected, then the case can be described as generalised, and if < 30% of teeth are affected, it can be described as localised [48]. This appears to be a reasonable approach to follow in general terms, but clinicians should not become too dogmatic in applying this threshold - as mentioned above, the diagnosis should take all factors into account.

It is important to note, however, that the descriptors of “localised” and “generalised” in the context of aggressive periodontitis have more specific connotations. Aggressive periodontitis is usually diagnosed in young (and otherwise healthy) adults in whom there is advanced periodontitis with rapid attachment loss and bone destruction, and a familial aggregation [52]. Localised aggressive periodontitis is probably not just a localised form of generalised aggressive periodontitis. In the case of localised aggressive periodontitis, there is a characteristic localised first molar/incisor presentation. The case definition described in the consensus report on aggressive periodontitis in the 1999 classification [52] describes interproximal attachment loss on at least two permanent teeth, one of which is a first molar, and involving no more than two teeth other than first molars and incisors. Other features include circumpubertal onset, and a robust serum antibody response to the infecting agents. In the case of generalised aggressive periodontitis, the consensus report refers to interproximal attachment loss that affects at least three permanent teeth other than first molars and incisors [52]. Other features of generalised aggressive periodontitis are that it usually affects people < 30 years of age (but may affect older individuals), there is a poor serum antibody response to the infecting agents, and there is a pronounced episodic pattern of tissue destruction [52].

With regards to severity of disease, this relates to the amount of inflammation (in the case of gingivitis) and the amount of attachment loss (in the case of chronic periodontitis). Terms such as “mild”, “moderate”, and “severe” are frequently used, being somewhat subjective, but also quite helpful clinically, as they are well understood by other clinicians. An assessment of severity based on mm of clinical attachment loss has been provided: 1-2 mm = slight, 3-4 mm = moderate, ≥ 5 mm = severe [48]. Again, this system is useful, but is sometimes not possible to be applied in a simple manner (for example, in cases with very variable attachment loss throughout the dentition). Radiographic bone loss is also useful for assigning descriptors to indicate severity of periodontitis: <1/3 bone loss = mild, 1/3 to ½ bone loss = moderate, and >½ bone loss = severe. It is important, however, to always take into consideration the full clinical picture when applying such descriptors, such as the age of the patient, presence of risk factors, degree of inflammation, probing depths, and pattern of bone loss (e.g. horizontal vs. vertical bone defects).

The diagnosis may therefore form a sentence that encapsulates the key features of the case, which may describe different disease states at different locations in the same mouth. Examples of possible diagnoses for different patients could be as follows:

• generalised severe gingivitis, with localised moderate chronic periodontitis (probing depths 4-5 mm) affecting interproximal sites at maxillary molars

• generalised moderate chronic periodontitis (probing depths 4-5 mm) with localised severe chronic periodontitis (probing depths 6-10 mm) at maxillary and mandibular molars

In many cases, it is also useful to list specifically the teeth affected by mild, moderate or severe disease, as this information, combined with the radiographic examination, will help to inform treatment decisions.

## Conclusions

Assessment and diagnosis of periodontal conditions that are amenable to prevention is a complex and challenging task. It is essential, however, to undertake periodontal screening in all our patients, given that the consequences of periodontal disease (attachment loss, alveolar bone loss, and ultimately, tooth loss), are largely irreversible. Key aspects relevant to the detection and diagnosis of periodontal conditions include:

• for individuals without evidence of periodontitis, periodontal screening using the CPI/BPE/PSR systems is essential, both as part of the initial assessment of new patients and as part of their regular ongoing care;

• for individuals with periodontitis, full periodontal assessment is required. This includes full mouth probing and bleeding on probing assessments, together with assessment of other relevant parameters such as recession, tooth mobility and furcation involvement;

• radiographic assessment is driven by the clinical situation, and is required to assess alveolar bone levels in patients with periodontitis;

• for patients with treated periodontitis in the maintenance phase of care (supportive periodontal therapy), full periodontal assessment is required on an ongoing basis to ensure that any evidence of disease progression is detected;

• risk assessment and management (e.g. in relation to factors such as smoking and diabetes) should form a central component of periodontal therapy.

## List of abbreviations used

BOP: Bleeding on Probing; BPE: Basic Periodontal Examination; CPI: Community Periodontal Index; CPITN: Community Periodontal Index of Treatment Need; FGDP: Faculty of General Dental Practice; LPS: Lipopolysaccharide; NHANES: National Health and Nutrition Examination Survey; PSR: Periodontal Screening Record; WHO: World Health Organisation.

## Competing interests

Professor Preshaw received funding from Colgate Palmolive to attend and present at the Prevention in Practice conference. This was provided in the form of flights, hotel accommodation and subsistence. No one from the Colgate Palmolive Company was involved in the production, assessment or peer review of the manuscript nor was it submitted to them for approval prior to publication. Professor Preshaw has also received funding from Colgate Palmolive on an ad hoc basis for presentation of clinical periodontal and scientific information at conferences.

## Declarations

This article has been published as part of *BMC Health Services Research* Volume 15 Supplement 1, 2015: Proceedings of Prevention in practice - making it happen. The full contents of the supplement are available online at http://www.biomedcentral.com/bmchealthservres/supplements/15/S1. Publication charges for this supplement were funded by Colgate Palmolive.
